# Association of Dry Eye Diseases and Auditory Sensitivity

**DOI:** 10.3390/jcm11144171

**Published:** 2022-07-18

**Authors:** Kyung Wook Kim, Jin Sun Hwang, Jiwon Chang, Young Joo Shin

**Affiliations:** 1 Department of Ophthalmology, Hallym University Medical Center, Hallym University College of Medicine, Seoul 07442, Korea; ddubogy@hallym.or.kr (K.W.K.); hotsayme@naver.com (J.S.H.); 2 Hallym BioEyeTech Research Center, Hallym University College of Medicine, Seoul 07442, Korea; brune@hallym.or.kr; 3 Department of Otorhinolaryngology, Hallym University Medical Center, Hallym University College of Medicine, Seoul 07442, Korea

**Keywords:** dry eye, bilateral hearing loss, tinnitus, hearing threshold

## Abstract

This study aimed to evaluate the association between dry eye and inner ear diseases in a Korean population. Methods: Data from the Korean National Health and Nutrition Survey (KNHANES V, 2010–2012), a national cross-sectional health examination and survey, were collected by the Korea Centers for Disease Control and Prevention. The association between dry eye and inner ear disease was determined using the chi-square test and logistic regression analysis. The individuals were divided into two age groups (<60 and ≥60 years). Results: In total, 17,542 individuals (*n* = 11,932 in the <60 years group and *n* = 5610 in the ≥60 years group) were enrolled. After adjusting for confounding factors, the logistic regression model revealed that the associated factors were dizziness and loss of balance experience (OR, 1.315; 95% CI, 1.143–1.513), self-awareness of abnormal voice (OR, 1.372; 95% CI, 1.120–1.679), subjective hearing discomfort (OR, 1.278; CI, 1.084–1.506), and tinnitus (OR, 1.265; 95% CI, 1.101–1.453). The inversely associated factor for dry eye was bilateral hearing loss (OR, 0.497; 95% CI, 0.367–0.672). The hearing threshold was lower in the dry eye group than in the non-dry eye group (*p* < 0.05). Conclusions: Tinnitus was associated with dry eye and bilateral hearing loss was inversely associated with dry eye. These results suggest that hypersensitivity of the senses and nerves, which is neuropathic hyperesthesia, is one of the main mechanisms of dry eye. Treatment of neuropathy may help in treating dry eye associated with dizziness or tinnitus.

## 1. Introduction

Dry eye is a common disease, with a prevalence of 5–50% [1,2]. Dry eye is characterized by symptoms such as pain, burning sensation, cold sensitivity, and foreign body sensation, which are caused by tear film instability, decreased tear secretion, and increased tear evaporation [2,3]. Although inflammation, oxidative stress, and endoplasmic reticulum stress have been reported to contribute to dry eye, neuropathic theory has been proposed as one of the pathogeneses of dry eye [4,5,6,7]. Ocular neuropathic pain is a condition in which pain is felt with or without normal stimuli [8]. Age-related dry eye disease is associated with corneal nerve fiber attrition, caused by an increased sensitivity or corneal hyperalgesia to tear evaporation [4,9,10]. The subepithelial nerves of the cornea are thicker [11], and the corneal sub-basal nerves show increased tortuosity, number of beadings, and width in dry eye patients [12], which supports the neuropathic theory of dry eye.

Neuropathic mechanisms include hypersensitivity of the peripheral nerve to normal sensations, experiencing non-existent sensations, and perception of sensations to be suppressed in the central nervous system [13,14]. Tinnitus is the perception of sound in the absence of an external sound stimulus [15]. It has neuropathic causes and is similar to neuropathic pain [16]. Conversely, hearing loss is a disease that entails the development of insensitivity to external sounds and has rarely neuropathic causes [17,18]. Although the relationship between hearing loss and glaucoma, diabetic retinopathy, and age-related macular degeneration has been described [19], the relationship between inner ear diseases and dry eye has not been reported. However, dry eye may be associated with hearing sensitivity or tinnitus, because both dry eye and tinnitus are associated with neuropathic sensations. In this study, we evaluated the relationship between dry eye and hearing loss and tinnitus.

## 2. Materials and Methods

The Korean National Health and Nutrition Examination Survey (KNHANES) was approved by the Institutional Review Board (IRB) of the Korean Centers for Disease Control and Prevention, and all participants provided written informed consent. This study adhered to the tenets of the Declaration of Helsinki and was approved from IRB approval by the institutional review board of Hallym University Kangnam Sacred Heart Hospital.

For this cross-sectional, population-based study, we used data from the Korean National Health and Nutrition Survey V (2010–2012), a series of cross-sectional surveys of nationally representative samples of the civilian Korean population, conducted annually to assess the health and nutritional status of the South Korean population. To obtain representative samples, KNHANES uses a stratified, multistage, cluster probability sampling design according to geographical area, age, and sex. For the health interview survey, a trained interviewer asked questions directly to individuals aged ≥19 years old. This study included 17,542 adults (7434 men and 10,108 women) aged ≥19 years who met the eligibility criteria, completed a questionnaire regarding independent risk factors, and underwent slit-lamp examinations. 

**Figure 1:**
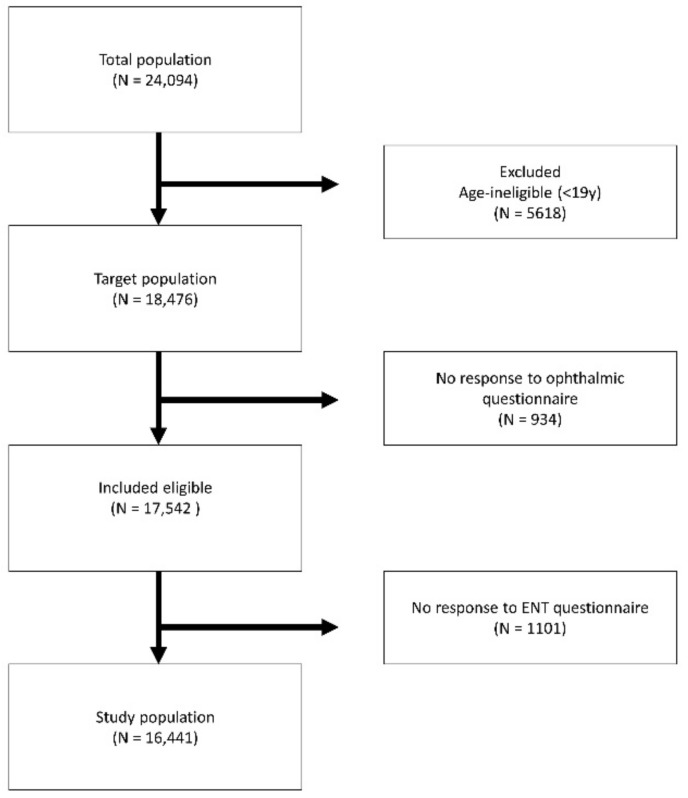
Flowchart of the study population KHANES V.

Dry eye was evaluated by ophthalmologist. The subjects were asked whether they were diagnosed with dry eye by an ophthalmologist as like the interview about whether thyroid disorder or heart diseases was diagnosed. Interview was conducted by ophthalmologists. Risk factor analysis using this type of dry eye diagnosis by KHANES has been reported in a lot of studies [20,21,22,23]. Data collected from the 2010 to 2012 KNHANES were analyzed in the present study. Unmatched or untested responses (untested) were excluded (Figure 1). The population was divided according to age: young (<60 years) or older (≥60 years). The participants were asked whether they had been diagnosed with dry eye, dizziness, loss of balance, severe dizziness, falls, self-awareness of abnormal voice, period of voice abnormality, subjective hearing discomfort, hearing aid or artificial cochlear implant use, tinnitus, and discomfort due to the tinnitus. The ears of participants were examined using a 4 mm 0° angled rigid endoscope attached to a charge-coupled device camera to find tympanic membrane perforation or cholesteatoma, retraction pocket, otitis media with effusion or abnormal external auditory canal. Only one ear was evaluated to exclude the possibility of statistical duplication.

Pure-tone audiometric testing was conducted using an SA 203 audiometer (Entomed, Malmö, Sweden). All audiometric tests were performed in a soundproof booth inside a mobile bus reserved for the KNHANES under the supervision of an otolaryngologist. The lowest level at which the participant responded to 50% of the pure tone was set as the threshold. The tested frequency ranges were 0.5, 1, 2, 3, 4, and 6 kHz. Hearing loss was categorized as unilateral or bilateral. Unilateral hearing loss was defined as hearing loss at a threshold of ≥25 dBHL in the ear with worse hearing, and bilateral hearing loss as hearing loss at ≥25 dBHL in the ear with better hearing.

Statistical analyses were performed using SPSS Version 27.0 (SPSS Inc., IBM Software, Portsmouth, UK), and two-sided *p*-values less than 0.05 were considered statistically significant. The chi-square test was used to compare discrete variables between the groups. The percentage (%) differences were calculated as the absolute value of the change in value divided by the average of the two numbers, all multiplied by 100. To estimate the odds ratios (ORs) of dry eye and potential factors, we conducted logistic regression analyses using a generalized linear model for a complex survey design. The ORs and 95% confidence intervals (CIs) were calculated in the following ways: confounder adjustment for age and sex.

## 3. Results

### 3.1. General Characteristics

The characteristics of the study population are shown in Table 1. The Pearson χ2 test showed significant differences in dizziness and loss of balance (*p* < 0.001), self-awareness of abnormal voice (*p* < 0.001), subjective hearing discomfort (*p* = 0.002), tinnitus (*p* < 0.001), tympanic membrane of the left ear (*p* = 0.030), and bilateral hearing loss (*p* < 0.001) in dry eye.

### 3.2. Factors Associated with Dry Eye

Table 2 shows the logistic regression analysis between dry eye and potential risk factors, adjusted for age and sex. The analysis showed that the risk factors for dry eye included dizziness and loss of balance (OR, 1.355; 95% CI, 1.188–1.546), self-awareness of abnormal voice (OR, 1.453; 95% CI, 1.222–1.727), subjective hearing discomfort (OR, 1.294; 95% CI, 1.118–1.498), and tinnitus (OR, 1.381; 95% CI, 1.236–1.543). Bilateral hearing loss (OR, 0.597; 95% CI, 0.452–0.790) was a protective factor against dry eye.

In the logistic regression analysis between dry eye and potential risk factors adjusted for age, sex, dizziness, loss of balance, self-awareness of abnormal voices, subjective hearing discomfort, tinnitus, and bilateral hearing loss (Table 3), the risk factors for dry eye included dizziness and loss of balance (OR, 1.315; 95% CI, 1.143–1.513), self-awareness of abnormal voices (OR, 1.372; 95% CI, 1.120–1.679), subjective hearing discomfort (OR, 1.278; 95% CI, 1.084–1.506), and tinnitus (OR, 1.265; 95% CI, 1.101–1.453). The protective factor for dry eye was bilateral hearing loss (odds ratio (OR), 0.497; 95% confidence interval (CI), 0.367–0.672). Table 4 shows the auditory thresholds for dry eye. They were lower in patients with dry eye at 3000 Hz or more in the right ear and at all frequency ranges in the left eye.

## 4. Discussion

Dry eye is mainly caused by decreased tear volume and increased evaporation [24]. Risk factors for dry eye, old age, female sex, menopause, obesity, rheumatoid arthritis, depression, and sleep disturbances have been described [1,25,26,27]. In this study, dizziness and loss of balance, self-awareness of abnormal voice, and tinnitus, which are associated with the inner ear [28], were identified as new risk factors. These results suggest that abnormalities in the inner ear are related to dry eye. The cochlea of the inner ear comprises a hollow bone shaped like a snail and is divided into two chambers by a membrane [29]. The chambers of the cochlea are filled with fluid, which vibrates when sound comes in, and 30,000 tiny hairs [29]. Fluid composition is delicately controlled, and changes in surface tension or composition of this fluid may cause abnormal signals or inflammation [30], which is reminiscent of the relationship between tears and the ocular surface.

Diseases of the inner ear, including dizziness, loss of balance, self-awareness of abnormal voice, and tinnitus, may contribute to the pathogenesis with dry eye. Dizziness and loss of balance indicate disturbances or inflammation of the inner ear, such as labyrinthitis or vestibular neuritis [31,32]. Neuroinflammation and neuropathy, which are associated with dizziness, reportedly play critical roles in dry eye [33,34,35]. The neutrophil-to-lymphocyte ratio, a systemic inflammatory marker, is high in both peripheral vertigo and dry eye [36,37,38,39,40,41,42]. Self-awareness of an abnormal voice may be due to the fact of vocal problems, conduction disorders, or inner-ear problems. Recently, it was reported that laryngopharyngeal reflux disease is associated with dry eye and pepsin was detected in tears [43]. The laryngopharyngeal reflux disease may contribute to self-awareness of an abnormal voice as laryngopharyngeal inflammation.

The possible mechanisms of the association between dry eye and tinnitus can be suggested as follows. First, both dry eye and tinnitus may have common causes, such as neuropathy or neuroinflammation. Neuropathic causes are important in dry eye [35,44,45], and tinnitus is associated with chronic neuropathic pain [46,47]. Chronic dry eye induces corneal hypersensitivity, neuroinflammatory responses, and synaptic plasticity in the mouse trigeminal brainstem [34]. Tinnitus may be caused by abnormal neural activity generated in the brain without the involvement of the ear [15,30]. Phantom sensations in tinnitus are similar to those in ocular neuropathic pain. Second, sleep disturbance due to the fact of tinnitus may have caused dry eye, because sleep disorders are an important risk factor for dry eye [48,49,50]. Sleep apnea accompanied with laryngopharyngeal reflux also may affect to dry eye [43,51]. Sleep disorders are associated with both tinnitus and dry eye [48,49,50,52,53]. Third, tinnitus can lead to high levels of stress, anxiety, and depression [54,55], and medication for these psychological problems may cause dry eye [56]. Fourth, the systemic hypersensitivity of sensations may result in both dry eye and tinnitus. In this study, the auditory threshold was lower in dry eyes, especially at high frequencies, indicating that patients with dry eye syndrome were more sensitive to sound. In dry eye, the sensory threshold is lowered, resulting in sensory and nerve hypersensitivity and potentially causing abnormal nociception and neuropathic symptoms on the ocular surface.

Conversely, bilateral hearing loss was inversely associated with dry eye. The causes of bilateral hearing loss include age, noise exposure, heredity, and medication [57]. The neuropathic causes of bilateral hearing loss are rare [18]. Unilateral hearing loss was not associated with dry eye, which may be because unilateral hearing loss may be associated with head trauma or injury, acoustic neuroma, or viral or bacterial infections [57]. 

The limitations of this study are that this study was conducted in Korean adults older than 19 years and dry eye diagnosis was assessed by interviews without objective examination of ocular surface and tear film. However, dry eye was evaluated by ophthalmologists. There have been a lot of studies reporting risk factor analysis using this type of dry eye diagnosis by KHANES [20,21,22,23]. Further study is necessary to include cross-sectional study design or cohort study design.

## 5. Conclusions

Tinnitus is associated with dry eye, whereas bilateral hearing loss is inversely associated with dry eye. The hearing threshold was lower in the dry eye group. These results suggest that hypersensitivity of the senses and nerves, which is a neuropathic hyperesthesia, is the primary mechanism of dry eye. Treatment of neuropathy may help treat dry eye syndrome associated with dizziness or tinnitus.

## Figures and Tables

**Table 1:** Clinical characteristics according to dry eye in the study population.

	Non-Dry Eye	Dry Eye	% Difference	*p*-Value
N (%)	15,720 (89.6%)	1822 (10.4%)		
Age				0.015 *
<60 years	10,734 (90.0%)	1198 (10.0%)		
≥60 years	4986 (88.9%)	624 (11.1%)	1.23	
Dizziness and loss of balance				<0.001 *
No	8566 (90.3%)	923 (9.7%)		
Yes	2269 (85.8%)	377 (14.2%)	5.11	
Severe dizziness				0.144
No	703 (85.3%)	121 (14.7%)		
Absent now, but present within a year	1206 (85.1%)	211(14.9%)	0.23	
Yes	360 (88.9%)	45 (11.1%)	4.13	
Fall				0.183
No	1969 (85.4%)	336 (14.6%)		
Yes	300 (88.2%)	40 (11.8%)	3.22	
Self-awareness of abnormal voice				<0.001 *
No	14,416 (89.9%)	1612 (10.1%)		
Yes	990 (85.3%)	171 (14.7%)	5.25	
Period of voice abnormality				0.447
<3 weeks	404 (86.3%)	64 (13.7%)		
>3 weeks	584 (84.6%)	106 (15.4%)	1.99	
Subjective hearing discomfort				0.002 *
No uncomfortable	13,071 (89.9%)	1462(10.1%)		
A little uncomfortable	1821 (87.3%)	266 (12.7%)	2.93	
Very uncomfortable	466 (90.1%)	51 (9.9%)	0.22	
Unable to hear	48 (92.3%)	4 (7.7%)	2.63	
Hearing aid or artificial cochlear implant use				0.404
No	2152 (87.7%)	302 (12.3%)		
Rarely used	52 (92.9%)	4 (7.1%)	5.76	
Yes	130 (89.7%)	15 (10.3%)	2.25	
Tinnitus				<0.001 *
No	11,932 (90.4%)	1260 (9.6%)		
Yes	3431 (86.8%)	522 (13.2%)	4.06	
Unable to remember	43 (97.7%)	1 (2.3%)	7.76	
Discomforts of life due to the fact of tinnitus				0.469
No	2246 (87.2%)	330 (12.8%)		
Annoying	1057 (86.3%)	168 (13.7%)	1.04	
Difficult to sleep	128 (84.2%)	24 (15.8%)	3.5	
Unilateral hearing loss				0.594
No	13,306 (89.5%)	1,563 (10.5%)		
Yes	1126 (90.0%)	125 (10.0%)	0.56	
Bilateral hearing loss				<0.001 *
No	13,645 (89.3%)	1629 (10.7%)		
Yes	787 (93.0%)	59 (7.0%)	4.06	

*p* Values were determined using the chi-squared test for categorical variables. * *p* < 0.05.

**Table 2:** Factors that affect the occurrence of dry eye after adjusting for age (60 years) and gender.

	OR	95% CI	*p*-Value
Dizziness and loss of balance experience			
No	1.0 (ref)		
Yes	1.355	1.188–1.546	<0.001 *
Severe dizziness			
No	1.0 (ref)		
Absent now, but present within a year	1.347	0.932–1.945	0.113
Yes, present	0.996	0.780–1.270	0.972
Fall			
No	1.0 (ref)		
Yes	0.793	0.557–1.129	0.197
Self-awareness of abnormal voice			
No	1.0 (ref)		
Yes	1.453	1.222–1.727	<0.001 *
Period of voice abnormality			
<3 weeks	1.0 (ref)		
>3 weeks	1.179	0.840–1.655	0.341
Subjective hearing discomfort			
No uncomfortable	1.0 (ref)		
A little uncomfortable	1.294	1.118–1.498	0.001 *
Very uncomfortable	1	0.738–1.355	1
Unable to hear	0.763	0.272–2.136	0.606
Hearing aid or artificial cochlear implant use			
No	1.0 (ref)		
Rarely used	0.944	0.541–1.647	0.84
Yes	0.621	0.221–1.747	0.367
Tinnitus			
No	1.0 (ref)		
Yes	1.381	1.236–1.543	<0.001 *
Unable to remember	0.201	0.028–1.468	0.114
Discomforts of life due to the fact of tinnitus			
Not uncomfortable	1.0 (ref)		
Annoying	1.317	0.832–2.086	0.24
Difficult to sleep	1.186	0.741–1.899	0.478
Unilateral hearing loss			
No	1.0 (ref)		
Yes	0.946	0.775–1.153	0.581
Bilateral hearing loss			
No	1.0 (ref)		
Yes	0.597	0.452–0.790	<0.001*

*p* Values were determined by binominal logistic regression analysis. * *p* < 0.05.

**Table 3:** Factors that affect dry eye after adjusting for age, gender, dizziness, self-awareness of abnormal voice, subjective hearing discomfort, tinnitus and bilateral hearing loss.

	OR	95% CI	*p*-Value
Dizziness and loss of balance	1.315	1.143–1.513	<0.001 *
Self-awareness of abnormal voice	1.372	1.120–1.679	0.001 *
Subjective hearing discomfort			
No uncomfortable			
A little uncomfortable	1.278	1.084–1.506	0.003 *
Very uncomfortable	1.165	0.817–1.663	0.399
Unable to hear	1.502	0.513–4.399	0.458
Tinnitus			
No			
Yes	1.265	1.101–1.453	0.001 *
Unable to remember	0.33	0.044–2.469	0.28
Bilateral hearing loss	0.497	0.367–0.672	<0.001 *

*p* Values were determined by binominal logistic regression analysis. * *p* < 0.05.

**Table 4:** Auditory thresholds according to dry eye.

	Non-Dry Eye	Dry Eye	*p*-Value
N	14,728	1713	
Age (Y)	50.63 ± 16.23	51.72 ± 15.90	0.009 *
Right ear			
500 Hz (dB)	36.34 ± 138.81	30.47 ± 118.83	0.058
1000 Hz (dB)	34.39 ± 139.16	28.66 ± 119.17	0.065
2000 Hz (dB)	37.19 ± 138.95	31.52 ± 119.02	0.067
3000 Hz (dB)	40.94 ± 138.94	34.14 ± 119.15	0.028 *
4000 Hz (dB)	45.30 ± 138.70	36.44 ± 116.81	0.004 *
6000 Hz (dB)	55.49 ± 137.65	47.74 ± 118.17	0.012 *
Mean (dB)	37.23 ± 138.88	31.21 ± 118.92	0.052
Left ear			
500 Hz (dB)	37.37 ± 138.66	31.24 ± 118.92	0.047 *
1000 Hz (dB)	33.94 ± 138.26	27.70 ± 119.20	0.044 *
2000 Hz (dB)	37.54 ± 139.01	31.05 ± 119.03	0.036 *
3000 Hz (dB)	42.08 ± 138.95	34.22 ± 119.16	0.011 *
4000 Hz (dB)	46.27 ± 138.68	37.28 ± 119.05	0.004 *
6000 Hz (dB)	57.23 ± 137.55	49.60 ± 118.16	0.013 *
Mean (dB)	37.75 ± 138.88	31.07 ± 118.89	0.031 *

*p* Values were determined by independent *t*-test. * *p* < 0.05.

## Data Availability

All the data utilized in this study are publicly available through the KNHANES website (http://knhanes.cdc.go.kr, accessed on 14 July 2022).
